# Mean weighted residuals reveal systematic overestimation of Bragg intensities in single-crystal diffraction

**DOI:** 10.1107/S1600576726003110

**Published:** 2026-05-08

**Authors:** Julian Henn, Piero Macchi, Toms Rekis

**Affiliations:** a DataQ Intelligence UG, Fichtelgebirgsstrasse 66, Germany; b Department of Chemistry, Materials and Chemical Engineering, Politecnico di Milano, Via Bassini 6, 20133, Milano, Italy; c Institute of Inorganic and Analytical Chemistry, Goethe University Frankfurt, Max-von-Laue Str. 7, 60438 Frankfurt am Main, Germany; The University of Western Australia, Australia

**Keywords:** systematic errors, rewarding errors, metrics, single-crystal diffraction

## Abstract

The mean value of weighted residuals (〈ζ〉) was analysed for 8424 published single-crystal X-ray data sets of crystals containing only light elements (C, H, N, O). A striking asymmetry was observed: 71.5% of data sets exhibit positive 〈ζ〉 values, occurring 2.5 times more often than negative values. This imbalance suggests systematic errors, with evidence pointing to a slight overestimation of observed intensities (*I*_obs_). Simulations and theoretical analysis show that such overestimation artificially lowers common data-quality metrics, including the popular merging factor *R*_merge_, the redundancy independent factor *R*_r.i.m._, the precision indicating factor *R*_p.i.m._, the weighted agreement factor *wR*(*F*^2^) and even atomic displacement parameters, creating a ‘rewarding error’ that may reinforce confirmation bias. Experimental data confirm these findings, as residual factors reach their minima for 〈ζ〉 > 0 rather than at zero. These results highlight the need for critical evaluation of data-processing strategies and caution against relying solely on conventional agreement factors as indicators of accuracy.

## Abstract 2

Analysis of more than 8000 published data sets demonstrates that systematic overestimation of observed intensities can deceptively improve common agreement factors and atomic displacement parameters, masking underlying deficiencies in data quality.

## 1. Introduction

Systematic errors in single-crystal diffraction are of general importance as the description and tracking of errors can be used to continuously improve the accuracy of the experiments, to validate data-acquisition and data-processing steps, to adjust parameter values in data-integration steps, to expose misconceptions, to validate new approaches and changes in hard- and software, and to improve correction procedures such as absorption and extinction models, as well as supporting modelling in challenging developing fields such as electron diffraction. In the recent past, the mean value of the weighted residuals 〈ζ〉, ζ = (*I*_obs_ − *I*_calc_)/σ(*I*_obs_), has been found to be a helpful data descriptor. A significant deviation from zero indicates the presence of systematic errors, which is frequently the case (Henn, 2019 ▸). The significance of the deviation from zero is calculated by dividing 〈ζ〉 by the standard deviation of the mean value σ(〈ζ〉). The standard deviation of the mean value is given by the square root of the unbiased sample variance over the number of reflections, σ(〈*I*_obs_〉) = [var(ζ)/*N*_obs_]^1/2^, with the unbiased sample variance 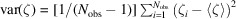. This definition for the significance of the mean value of the weighted residuals is analogous to the definition of the significance of a redundantly measured observed reflection, where the standard deviation of the mean value (as opposed to the standard deviation of the sample) is obtained by dividing the unbiased sample variance by the redundancy and taking the square root.

It was previously found that 〈ζ〉 tends to positive values. In a sample of 127 data sets published with *IUCrData* (https://iucrdata.iucr.org/x/), 52% of the data sets showed a significant positive deviation of the mean value of the weighted residuals from zero (Henn, 2019 ▸). These findings were later confirmed with an even larger sample of over 300 data sets from *IUCrData* (unpublished work). A positive shift of the residuals was connected earlier to several causes such as unrecognized low-energy contamination, unrecognized twinning and disorder problems (Domagala *et al.*, 2023 ▸).

It will be shown in this study how overestimation of observed intensities affects different metrics such as the merging *R* factor *R*_merge_, the redundancy independent merging factor *R*_r.i.m._, the precision indicating merging factor, *R*_p.i.m._ (Weiss, 2001 ▸) and the weighted agreement factor *wR*(*F*^2^): it leads to an artificial lowering in the studied metrics.

When aiming for high data quality, researchers may adjust various data-integration parameters. Success is then judged by monitoring commonly used metrics. These metrics can create the impression that quality is improving. In reality, after a certain point, the actual data quality begins to decline – even though the metrics continue to suggest improvement. Only after model refinement may these errors become apparent, for example by a systematic positive shift of the residuals, pro­vided these traces of systematic errors are actively searched for. This systematic shift may be so small for individual data sets that it remains well below the noise level; however, with many data sets in the sample, the shift is clearly exposed.

When certain systematic errors (such as a slight overestimation of observed intensities) lead to seemingly higher data quality with respect to certain metrics [such as *R*_r.i.m._, *R*_p.i.m._, *R*_merge_, *R*, *wR*(*F*^2^), *U*_*ij*_*etc*.], this is called a *rewarding error* with respect to the mentioned data quality metrics as it leads to an appreciated result. Rewarding errors are a particularly important class of errors since they meet the desired expectation of high data quality of the user. Therefore, they are less likely to be questioned (confirmation bias) and may occur frequently but may remain undetected for decades. When crystallographic software developers unintentionally and unknowingly fall for confirmation bias, this can also lead to undetected methodological issues.

The present work aims at confirming the tendency to positive residuals for a much larger sample of published data sets comprising only light elements (in order to minimize the impact of absorption correction errors discussed previously (Henn, 2025 ▸). Additionally, a possible explanation is offered by proposing that slight overestimation of *I*_obs_ on average is likely to be a cause of the shift of the residuals towards positive values.

## 2. The data

A total of 8424 crystallographic data sets containing only C, H, O and N were downloaded from the Crystallography Open Database (COD; Vaitkus *et al.*, 2023 ▸; Mesto *et al.*, 2013 ▸; Vaitkus *et al.*, 2021 ▸; Quirós *et al.*, 2018 ▸; Merkys *et al.*, 2016 ▸; Gražulis *et al.*, 2015 ▸; Gražulis *et al.*, 2012 ▸; Gražulis *et al.*, 2009 ▸; Downs & Hall-Wallace, 2003 ▸). The CIF tag _exptl_absorpt_process_details was used to determine the absorption correction processing software. Data processed with different releases of *SADABS* (Krause *et al.*, 2015 ▸), *SORTAV* (Blessing, 1987 ▸; Blessing, 1997 ▸; Blessing, 1995 ▸), Rigaku/Oxford Diffraction and Stoe & Cie software were included in the sample. The overwhelming majority of structure models use the independent atom model. It is known that the software versions may sometimes quote an older version of a program even when in fact the data were obtained with a newer version. No attempts were made to identify such cases. Most data sets were processed with one out of many releases of *SADABS* (*N* = 6781), with *SADABS* 1996 (*N* = 1919) having the largest share. Different releases of *CrysAlis PRO* [*CrysAlis PRO* Agilent releases from 2010 (*N* = 76), 2011 (*N* = 95), 2012 (*N* = 73), 2013 (*N* = 53) and 2014 (*N* = 80); *CrysAlis PRO* Oxford Diffraction releases from 2009 (*N* = 81) and 2010 (*N* = 71); *CrysAlis PRO* Rigaku OD 2015 (*N* = 72)], *CrysAlis RED* [*CrysAlis RED* Oxford Diffraction 2006 (*N* = 47), 2007 (*N* = 51), 2008 (*N* = 42) and 2009 (*N* = 83)] and *CrystalClear* [*CrystalClear* Rigaku MSC 2005 (*N* = 66) and *CrystalClear* Rigaku 2005 (*N* = 201)] add up to a total of 1091 Rigaku-associated data sets. Two releases of *SORTAV* follow [Blessing (1995 ▸) (*N* = 199) and Blessing (1997 ▸) (*N* = 33)], with a total of 232 data sets, and finally, *X-RED32* [Stoe & Cie 2002 (*N* = 235)].

The resolution limit as recalculated from θ_max_ and the wavelength ranges between 0.4476 and 1.1744 Å^−1^, with mean value 0.6402 Å^−1^ and median value 0.6276 Å^−1^. Fifty per cent of all data sets have a maximum resolution in the range 0.6024–0.6601 Å^−1^, with 25% of all data sets having a maximum resolution below and 25% above this range (see Table 1 ▸).

The number of observed intensities ranges between 314 from a low-temperature redetermination of metaldehyde (tetragonal space group *I*4) at 150 K [COD 2205445, Barnett *et al.* (2005 ▸)] and 38082 from a structure in monoclinic space group *P*2_1_/*n* containing a pyrene derivative C_34_H_37_N [COD 2240967, Thekku Veedu & Techert (2015 ▸)], measured at 100 K and refined as a non-merohedral twin. Only two data sets show a zero weighting scheme parameter *a* = 0 [*N*,*N*′-bis(3-methyl­phen­yl)succinamide dihydrate, monoclinic space group *P*2_1_/*c*, COD 2230904, Saraswathi *et al.* (2011 ▸); a polymorph of butobarbital, monoclinic space group *P*2_1_/*c*, COD 2016360, Gelbrich *et al.* (2007 ▸)]. A total of 1057 data sets show weighting scheme parameter *b* > 1; the largest values are 36.3175 [ethyl 4-butyl­amino-3-nitro­benzoate, monoclinic space group *C*2/*c*, COD 2222973, Narendra Babu *et al.* (2009 ▸)] and 38.7799 from a study of peptide nanotubes with flexible pores and disordered solvent [COD 2103455, Görbitz (2002 ▸)].

The maximum crystal dimension lies between 0.03 mm [COD 2230768, Ismiyev (2011 ▸); COD 2218750, Liang & Qu (2008 ▸); COD 2238399, Chetioui *et al.* (2013 ▸); COD 2022704, Aristov *et al.* (2023 ▸)] and 10.28 mm [benzohydrazide, C_25_H_29_N_3_O, obtained with Cu *K*α radiation at 296 K, COD 2234318, Bhat *et al.* (2012 ▸)]. The weighted agreement factor *wR*(*F*^2^) lies between 0.0483 [polymorph of *myo*-inositol, orthorhombic space group *Pna*2_1_, *T* = 180 K, COD 2212154, Khan *et al.* (2007 ▸)] and 0.3664 [hexamethylenetetramine, C_6_H_12_N_4_·2C_8_H_8_O_2_, monoclinic space group *P*2_1_/*n*, *T* = 173 K, COD 2104946, Lemmerer (2011 ▸)], with in total 19 data sets with *wR*(*F*^2^) > 0.3000 and 483 data sets with *wR*(*F*^2^) > 0.2000.

## 3. Mean and median of the weighted residuals increase simultaneously

**Figure 1:**
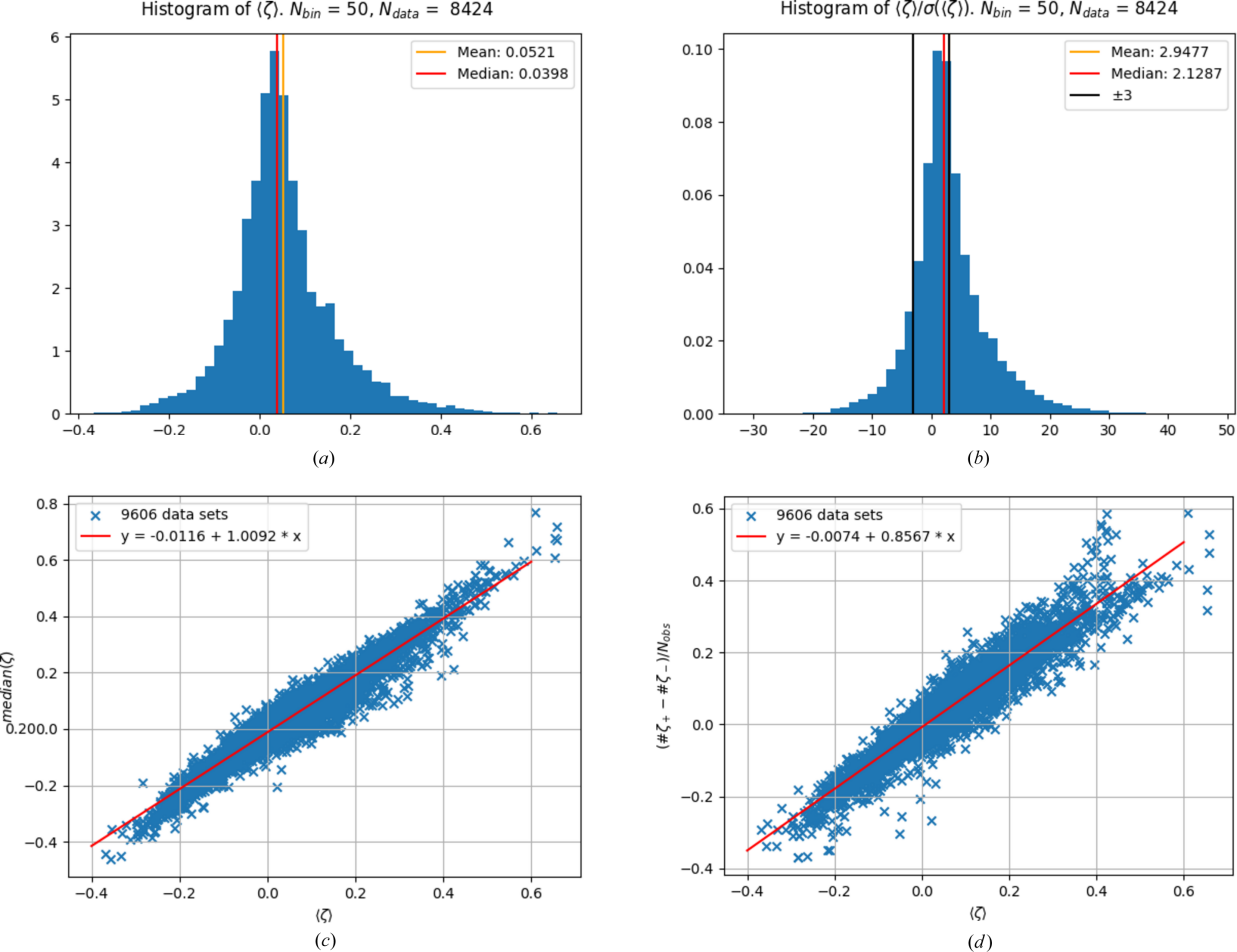
(*a*) The mean value of the weighted residuals is not at zero but at a slightly larger value of 0.0521. This appears to be a small value; however, plot (*b*) shows the histogram of the significance of the deviation from zero for 8424 analysed data sets. (*c*) The mean value of the weighted residuals is strongly correlated with the median of the weighted residuals. The 95% confidence intervals for the fit parameters are given by [−0.012, −0.011] for the constant and [1.003, 1.015] for the slope. (*d*) The shift of the residual distribution as a whole can also be described by the fraction of positive excess residuals. When this fraction is multiplied by 100 it gives the percentage of positive excess residuals. When the distribution of residuals for an individual data set is well centred at zero, the percentage of positive excess residuals is close to zero. The fitted parameters are given in the plot with the respective estimated standard deviations. The 95% confidence intervals for the fitted parameters are given by [−0.008, −0.007] for the constant and [0.850, 0.863] for the slope. Plots (*c*) and (*d*) together may indicate data-processing or -reduction problems. For details, see text.

Fig. 1 ▸(*a*) shows the histogram for the mean value of the weighted residuals 〈ζ〉 from the above-described sample of *N* = 8424 structures with light atoms C, H, O and N only. The brackets indicate averaging of the weighted residuals over individual data sets such that for every crystallographic data set one mean value of the weighted residuals 〈ζ〉 is obtained. The mean value over all data sets 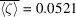, where the overline indicates now averaging over all data sets in the sample, is slightly larger than zero. This may appear to be a small value; however, Fig. 1 ▸(*b*) shows the histogram of the significance of the deviation from zero for the 8424 analysed data sets. A minority of only 45.45% are within the boundaries of ±3σ, as indicated by the black vertical lines; 41.79% of all data sets show a significance larger than plus three, and 12.76% show a significance less than minus three. Positive outliers appear 3.27-fold more frequently than negative outliers and in total the ‘outliers’ are the majority. In Fig. 1 ▸(*c*), the median of the weighted residuals is plotted against the mean value of the weighted residuals for each data set. A large correlation between these entities is obvious from the plot. This observation is a first hint that for each individual data set the distribution of weighted residuals is shifted *as a whole*, *i.e.* the mean value is *not* mainly determined by a few strong positive outliers. The shift of the residual distribution as a whole can also be described by the fraction of positive excess residuals [Fig. 1 ▸(*d*)]. A well centred Gaussian distribution results in approximately 50% positive residuals ζ > 0 := ζ_+_ and 50% negative residuals ζ < 0 := ζ_−_. The difference between the integer numbers of positive and of negative residuals *#*ζ_+_ − *#*ζ_−_ divided by the total number of weighted residuals *N*_obs_ (called ‘the fraction of positive excess residuals’) is in this case a number close to zero and is accidentally sometimes slightly larger and sometimes slightly smaller than zero. But the number of positive excess residuals increases with increasing mean value of the weighted residuals 〈ζ〉, which confirms and quantifies the observation from plot (*c*) that the shift of the distribution of weighted residuals is driven mainly by shifting the distribution as a whole, rather than by strong outliers.

When the 483 data sets with *wR*(*F*^2^) > 0.2000 are excluded, the following values result: 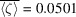 (to be compared with 0.0521 for all data sets), median〈ζ〉 = 0.0384 (to be compared with 0.0398 for all data sets), 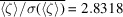 (to be compared with 2.9477) and median[〈ζ〉/σ(〈ζ〉)] = 2.0484 (to be compared with 2.1287), *i.e.* the values are all slightly reduced, but the qualitative picture does not change.

### 3.1. Over- and underestimation of *I*_obs_ by δ

When *I*_obs_ denotes the reflection intensity in the reflection input file, *I*_true_ denotes the (unknown) true intensity excluding random noise, ±Δ denotes random noise and δ denotes a constant systematic offset for all reflections, *e.g.* from a calibration error, and when no other errors apply, the resulting intensity is given by 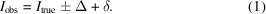
The symbol ±Δ was used to emphasize the stochastic nature of noise with equal probabilities for positive and for negative fluctuations. Its amplitude is characterized by the estimated standard uncertainty of the observed reflection s.u.(*I*_obs_) in the reflection input file. Stochastic noise is not affected by a constant shift of origin. With this notation, when δ = 0 for all reflections, the observed intensity is unbiased with respect to the true intensity when averaging over the noise: 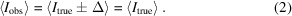
When, in contrast, a systematic offset δ ≠ 0 applies, 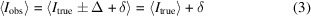
and the observed intensity is not unbiased anymore. It is affected by a systematic shift δ.

A constant and for all reflections equal positive or negative offset δ may be seen as the extreme case of a whole class of systematic errors where the origin for only a subgroup of observed intensities, for example from resolution or exposure time batches, is shifted (origin drift) or where non-linearities in area detectors lead to spatial or intensity-dependent origin drifts. Distinct spatial inhomogeneities in detector responses were reported earlier (Paciorek *et al.*, 1999 ▸; Pflugrath, 1999 ▸; Dudka, 2018 ▸). Insufficient or missing absorption correction procedures may affect specifically those reflections with the longest path through the crystal, though depending on the linear absorption coefficient of the crystal. Time-dependent drifts of the origin may occur from decreasing or fluctuating beam intensity, crystal decay, or just insufficient or missing correction for changing irradiated crystal volume. These errors may lead to different individual shifts depending on the coordinates of the detecting pixel in the detector (*x*_det._, *y*_det._), resolution, intensity, beam profile or exposure time, 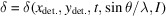, and on geometry parameters.

All of the mentioned errors may result in an average shift 〈δ〉 of the reflection intensities. So all of these systematic errors may be kept in mind when only a constant value δ is discussed for simplification as it can be regarded as the total net effect of individual errors 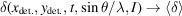. To further simplify the discussion, all of these errors are summarized under the keywords ‘overestimation’ and ‘underestimation’ of the observed intensity. Fig. 1 ▸ indicates a dominance of overestimation of *I*_obs_, so the focus is on overestimation.

A slight – but systematic – overestimation from, say, data-integration steps would not necessarily be visible in the individual data set, where other systematic errors may overlie and mask this specific small error. Additionally, a slight over- or underestimation *I*_obs_ = *I*_true_ ± Δ + δ would easily explain the strong correlation between median(ζ) and 〈ζ〉 as depicted in Fig. 1 ▸(*c*), as even small errors |δ_*hkl*_| < s.u.(*I*_obs,*hkl*_) in the abundant weak data would immediately lead to small linear positive and negative changes in the median of the residuals, just as observed in Fig. 1 ▸(*c*).

The working hypothesis is therefore from here on that small but systematic errors in *I*_obs_ are an important factor in explaining Fig. 1 ▸(*d*). Overestimation of *I*_obs_ on average explains the overall appearance of the plots in Figs. 1 ▸(*c*) and 1 ▸(*d*) by accounting for the linear increase in (*#*ζ_+_ − *#*ζ_−_)/*N*_obs_ with increasing 〈ζ〉.

But overestimation of *I*_obs_ on average also raises further questions: (i) Why would small errors more frequently lead to overestimation, 〈ζ〉 > 0, than to underestimation (negative shifts 〈ζ〉 < 0)? And (ii) would regular overestimation of *I*_obs_ not increase the residual factors and call for corrections in this way?

## 4. How overestimation of *I*_obs_ affects the residual factors

The last two questions have a surprising answer: The residual factors *decrease* when the observed intensities are slightly *over*estimated – and they tend to *increase* when the observed intensities are slightly *under*estimated. This may unconsciously incentivize overestimation of *I*_obs_ rather than underestimation in detector calibration experiments or when setting default data-integration parameter values. In the following section an example will be used to briefly discuss how and why the residuals factors are lower when the observed intensities *I*_obs_ are overestimated. In order to give more evidence that the rewarding behaviour of the agreement factors is not just a theoretical idea but a very tangible thing, a simulation is performed with artificial data to prove this point, and traces in data from experiments are presented to further substantiate the working hypothesis.

### 4.1. Overestimation of *I*_obs_ reduces *R*_merge_, *R*_r.i.m._ and *R*_p.i.m._

As an example, the merging *R* factor, *R*_merge_, which is also called *R*_sym_, is briefly discussed. The importance of the merging *R* factor lies in its availability, as it is very frequently given in published data sets. As a metric for data quality it has severe weaknesses (Diederichs & Karplus, 1997 ▸). These were solved with the redundancy independent merging *R* factor *R*_r.i.m._ and with the precision indicating merging *R* factor *R*_p.i.m._ (Weiss, 2001 ▸). These important descriptors should be included as standard.

The merging *R* factor is defined according to 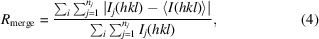
where *n*_*j*_ is the redundancy of the unique reflection *i* and where 〈*I*(*hkl*)〉 indicates the mean value over the redundantly measured reflection (Arndt *et al.*, 1968 ▸). The contribution from one arbitrarily chosen unique reflection *i* with redundancy *n*_*j*_ in the numerator is the sum 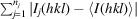. Suppose each individual measurement carries the same small constant error δ > 0. How does this affect the sum in the numerator? Obviously, it increases each individual reflection by the same amount 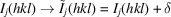, and as a consequence also the average value by the same amount: 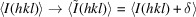. As these terms are subtracted, the sum remains unchanged: 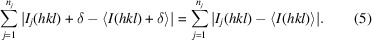
A short way to state this fact is ‘The sum in the numerator of equation (4) remains unchanged under a transformation *I*_*j*_(*hkl*) → *I*_*j*_(*hkl*) + δ’ – it is *invariant* under such a transformation. This is actually a trivial statement; it just means that the difference between numbers that are increased by the same amount remains unchanged. This holds also for each individual term in the numerator and thus for the numerator of equation (4) in total. The denominator of equation (4), however, is *not* invariant under such a transformation. It increases by *n*_*j*_δ for the unique reflection*i*: 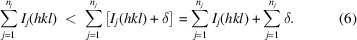
The last two equations taken together state that the merging *R* factor decreases when the observed intensities are overestimated by δ > 0 as the numerator is unchanged and the denominator increases in this case. The merging agreement factor responds ‘rewardingly’ to the systematic error of overestimated intensities when a low value is perceived as desirable (confirmation bias). This holds also when not all of the redundantly measured intensities are overestimated by the exact same value δ, which was only assumed to simplify the discussion; it also holds when the observed intensities are overestimated by different amounts δ_*j*_.^1^

So far, the discussion has assumed small errors δ. However, the conclusions also apply to large errors: indeed, the larger δ is, the smaller the merging *R* factor. In other words, the more the observed intensities are overestimated, the smaller *R*_merge_ gets.

The demonstrated behaviour of the merging *R* factor to reward overestimation of *I*_obs_ with lower values holds also for *R*_r.i.m._ and *R*_p.i.m._, as these differ from *R*_merge_ only in the factors [*n*_*i*_/(*n*_*i*_ − 1)]^1/2^ and 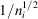, respectively, in the numerator.

### 4.2. Overestimation of *I*_obs_ reduces *wR*(*F*^2^), *R*_1_ and atomic displacement parameters

For the weighted agreement factor 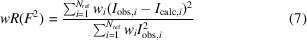
(with weights *w_i_* and where *N*_ref_ is the number of reflections included in the refinement) and for the conventional *R* factor 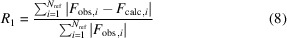
one cannot expect exactly the same results, as the structure model is involved in these cases in the form of *I*_calc_ or structure factor *F*_calc_. This is in contrast to *R*_merge_, *R*_r.i.m._ and *R*_p.i.m._ where the observed entities are not compared with calculated entities but only with other observed entities. For the weighted agreement factor and the conventional agreement factor it is reasonable to assume that overestimation of the observed intensity initially also lowers the respective residual factor, until, at some point, the difference between the weakest observed and calculated entities starts to increase the residual sum at a rate that is larger than the rate of increase of the denominator.

**Figure 2:**
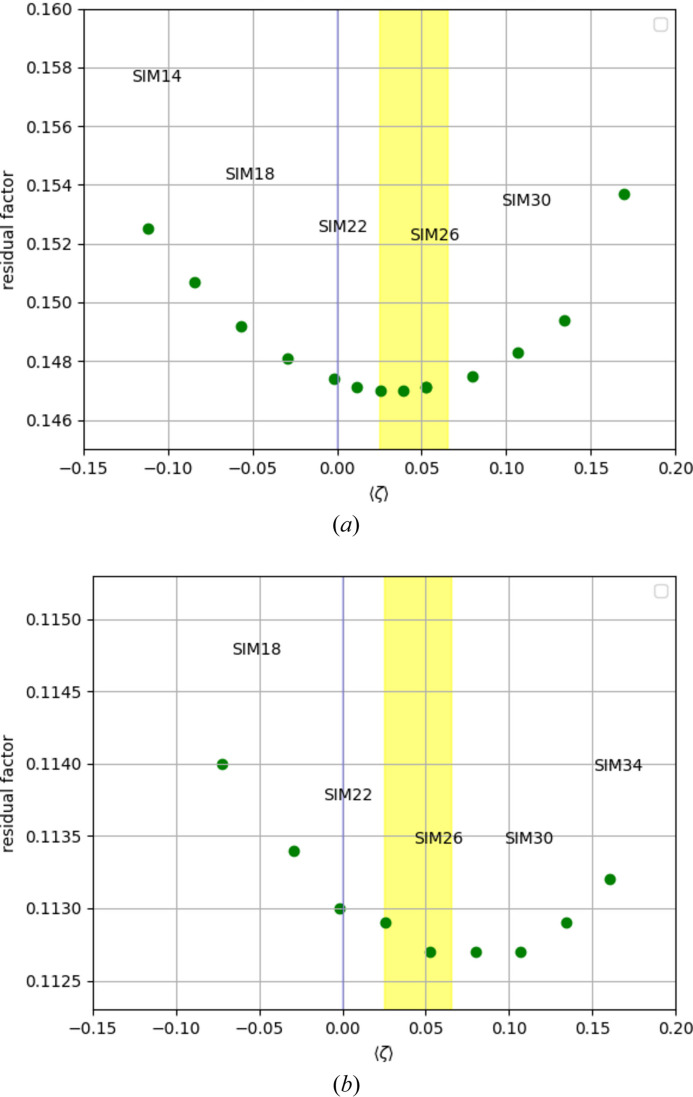
Simulations with artificial data. A calibration error is simulated by adding for each simulation a different constant amount δ to all observed intensities. The added amount is small in the sense that (i) it is smaller than 2 times the smallest value of σ(*I*_obs_) and (ii) model refinement against the simulated data results in weighting scheme parameters *a* = 0 and *b* = 0 for SIM 14–SIM 30. For the exact values of δ for each simulated data set see Table 2[App appa] in Appendix *A*[App appa]. (*a*) *wR*(*F*^2^) shows a minimum for slightly overestimated *I*_obs_. The minimum is at approximately 〈ζ〉 ≈ 0.03. (*b*) *R*_1_ shows a minimum at a larger positive value 〈ζ〉 ≈ 0.08. Both residual factors ‘reward’ overestimation of *I*_obs_.

In order to prove that the weighted agreement factor is initially decreasing as a response to a slight overestimation of *I*_obs_, a simulation was performed with artificial data (for more details about the simulations see Appendix *A*[App appa]). In the simulation we know the exact true values of the observed intensities, which are never known in an experiment. Additionally, the exact error δ is known, as are the true model parameter values. For the simulation, the calculated intensities were extracted after convergence of a refinement and written to a reflection input file. Gaussian random noise was added in proportion to the s.u.(*I*_obs_) values from the experiment. In order to perform the simulation at a level of the weighted agreement factor that approximately compares with average experimental values, the noise was chosen to be 4 times s.u.(*I*_obs_). A number of such reflection input files were generated. Different values δ were added in incremental steps in addition to the noise for different artificial data sets. The same starting model was refined against each of these resulting artificial reflection input files. The resulting residuals factors *wR*(*F*^2^) and *R*_1_ are plotted in Figs. 2 ▸(*a*) and 2 ▸(*b*), respectively, against the mean value of the weighted residuals 〈ζ〉. The lowest residual values are not attained for 〈ζ〉 = 0, as one might expect naively, but for 〈ζ〉 > 0. This proves that *wR*(*F*^2^) and *R*_1_ are rewarding overestimation of *I*_obs_ similarly to *R*_merge_, *R*_r.i.m._ and *R*_p.i.m._. The main effect of overestimation of *I*_obs_ on the structure model as obtained from the simulation is a systematic reduction in the atomic displacement parameter *U*_eqiv_. As an example, the true value for the chlorine atom in the simulation described in Appendix *A*[App appa] is *U*_eqiv_ = 0.05793. Increasing *I*_obs_ until 〈ζ〉 = 0.1071 leads to a reduction to *U*_eqiv_ = 0.05729, which corresponds to 2.56 standard deviations. The reduction in *U*_eqiv_ is systematic for all atoms and corresponds on average to 0.92 standard deviations.

## 5. Are these findings in accordance with the experimental data?

The theoretical considerations and the simulations from the previous sections showed that the residual factors *R*_merge_, *R*_r.i.m._ and *R*_p.i.m._, *wR*(*F*^2^), and *R*_1_ are lower when the true intensity is slightly overestimated compared with the case where the observed intensity is unbiased with respect to the true intensity. For *R*_merge_, *R*_r.i.m._ and *R*_p.i.m._ (and all other residual factors with a similar structure), this is easy to demonstrate theoretically, as in these cases only observed entities enter the defining equations. The numerators in all of these equations are composed of sums of absolute differences from observed intensities and mean values thereof. These differences remain unchanged when the observed intensities are overestimated, whereas the denominators in all of these equations increase. This leads to a reduction of the residual factors in response. In the case of *wR*(*F*^2^) and *R*_1_, model-derived entities are involved. This changes the situation slightly, but not qualitatively, when only a small overestimation of *I*_obs_ is considered. These residual factors also decrease initially for overestimation of *I*_obs_. A qualitative difference is that they finally start to indicate the systematic error if the overestimation is sufficiently distinct, whereas for *R*_merge_, *R*_r.i.m._ and *R*_p.i.m._ this is not the case.

The theoretically derived and simulation-confirmed rewarding behaviour of the residual factors with respect to overestimation of *I*_obs_ may explain why there are so many more data sets with a positive shift of the mean value of the residuals compared with those with a negative shift, where, ideally, positive and negative shifts should be equally distributed and equally strong.

The answer to this riddle could lie in the fact that overestimation of *I*_obs_ is rewarded such that it remains undetected in the data (confirmation bias). As a consequence, this should be visible from the experimental data themselves.

**Figure 3:**
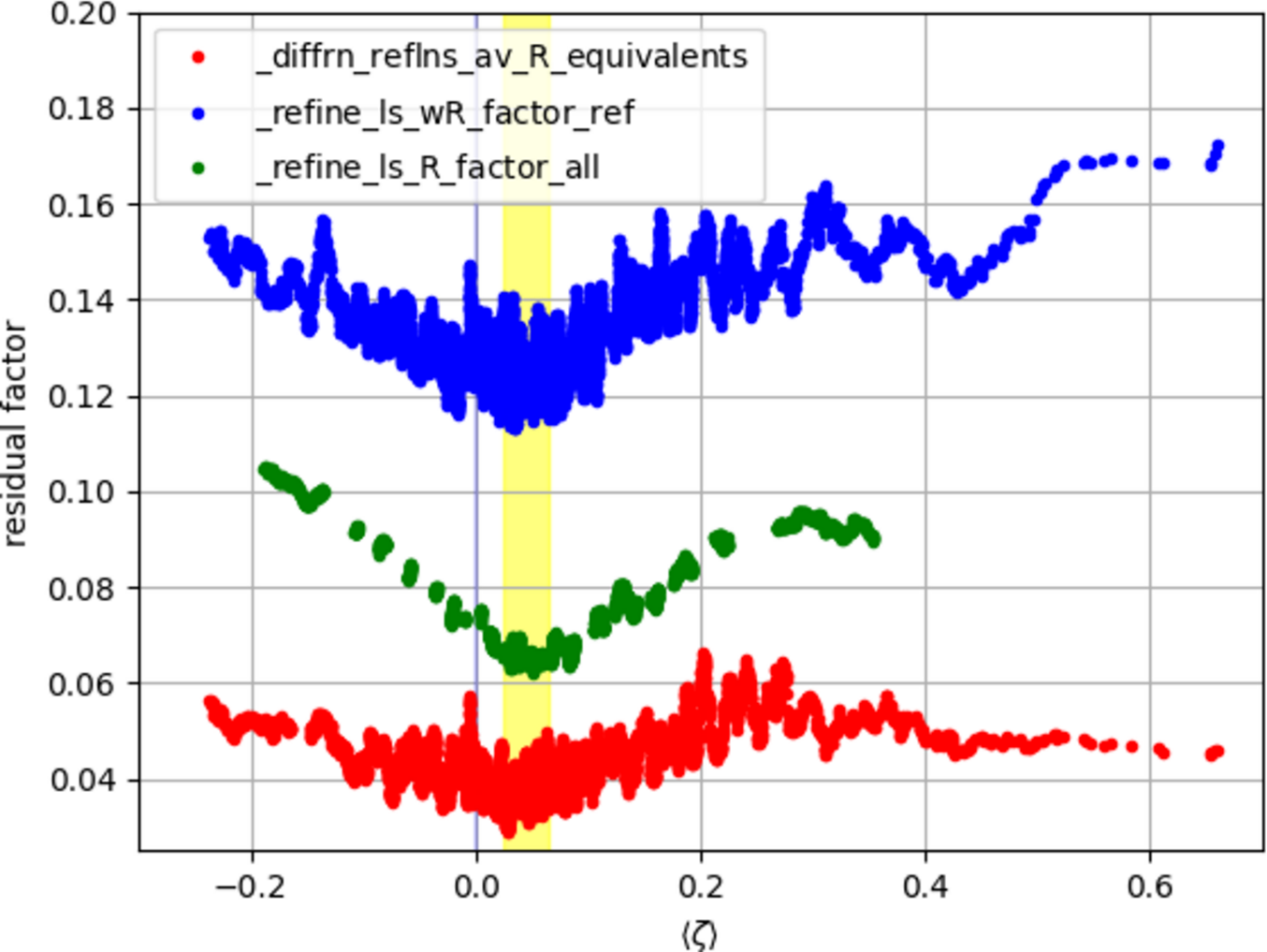
Residual factors (*y* axis) plotted as moving averages over a window of 50 consecutive data points as a function of 〈ζ〉 (*x* axis). Blue: _refine_ls_wR_factor_ref. Red: _diffrn_reflns_av_R_equivalents. Green: _refine_ls_R_factor_all. The plot contains empty spaces where individual data points were not available. The averaging continues only when 50 or more data sets were available in a row with the desired value due to the chosen window. Different residual factors show their minimum values also for 〈ζ〉 > 0. The common minimum area for the three residual factors is shown as a yellow stripe.

Fig. 3 ▸ shows the residual factors *R*_merge_ (_diffrn_reflns_av_R_equivalents, red), *wR*(*F*^2^) (_refine_ls_wR_factor_ref, blue) and *R*_1_ (_refine_ls_R_factor_all, green), plotted as moving averages (with a window of 50 consecutive data points) as a function of the mean value of the weighted residuals. The common area in which all three residual factors reach their respective minimum value is shown as a yellow stripe in the range 0.025 ≤ 〈ζ〉 ≤ 0.065. The same yellow stripe is also depicted in Fig. 2 ▸. This confirms again the overall slight overestimation of *I*_obs_.

## 6. Discussion and outlook

It was shown that the shift of the mean value of the residuals correlates strongly with the median of the residuals. This strong correlation is interpreted as a sign that the mean value of the weighted residuals is determined by a shift of the distribution of the residuals *as a whole* rather than by a small number of strong outliers. In other words, the abundant weak data in each data set may have a stronger influence on the mean value of the residuals than individual large outliers from other, more conventional errors, such as a slight unmodelled disorder or neglect of bonding density. This holds in particular when the abundant weak intensities are slightly over- or underestimated. The individual under- or overestimation of *I*_obs_ = *I*_true_ ± Δ ± δ may well be within the limits of noise 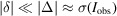 – it is virtually invisible on the level of the individual reflection – and may nevertheless influence the mean value of the weighted residuals as these many small but systematic errors δ accumulate. It was shown with the help of artificial data that slight overestimation of *I*_obs_ = *I*_true_ ± Δ + |δ| leads to a *lower* weighted agreement factor than would be obtained with the unbiased, true values of *I*_obs_ = *I*_true_ ± Δ. The true intensity is available in a simulation in contrast to an experiment. Note that overestimation of *I*_obs_ may already occur at the step of data integration and data processing.

It was furthermore shown that these ideas are not merely theoretical but are confirmed by experimental data. The minimum values for different residual factors are found in the sample of experimental data sets again for a slightly positive mean value of the weighted residuals – they are *not* centred around 〈ζ〉 = 0. This is taken as confirmation (i) that these residual factors respond in a rewarding way to slight overestimation of *I*_obs_ and (ii) that the rewarding behaviour may constitute an (unconscious) incentive for overestimation.

In the simulation presented in this study, under- and overestimation of *I*_obs_ was modelled by adding the same small amount ±*n*δ to each reflection. Different increments *n* resulted in different simulated data sets. This error is of course a simplified model of systematic errors in real experiments, where errors may be much more complicated. For discussion purposes, however, and for working out the consequences, it is a valid model. Also, detectors need to be calibrated. The calibration itself comes with an error, even if it is small. A calibration error offsets the origin for all reflections by the same value. Therefore, such an error may describe a real-world case quite accurately, even if it seems a little idealized and artificial at first glance.

A calibration error explains the simultaneous increase of the mean of the residuals with the number of positive excess residuals. The artificial lowering of agreement factors and of atomic displacement parameters by overestimation of *I*_obs_ explains why this error was overlooked for such a long time (confirmation bias). Overestimation of weak data in small-molecule single-crystal experiments was found and discussed earlier in the context of measurement strategies (Williams *et al.*, 2019 ▸) for low-exposure-time and high-resolution data. The current results indicate that the problem might be much more widespread.

### 6.1. Factors contributing to overestimation of *I*_obs_ from the perspective of metrics

Unintentionally allowing for a slight overestimation *I*_obs_ > *I*_Bragg_ of the observed intensities may unknowingly be supported by leading to more desirable results. A typical situation for confirmation bias is (i) lower residual factors *R*_merge_, *R*_r.i.m._, *R*_p.i.m._, *wR*(*F*^2^), *R*_1_ and similar and (ii) the atomic displacement parameters indicate smaller amplitudes, as briefly mentioned in Section 4.2. It is intuitive that small atomic displacement parameters are associated with high data quality as systematic errors tend to accumulate in displacement parameters by increasing them. For this reason, atomic displacement parameters from X-ray diffraction experiments are sometimes compared with results from neutron diffraction experiments [as an example see Chodkiewicz & Woźniak (2025 ▸)] in order to give evidence for the accuracy of the X-ray refinement. Neutron diffraction experiments have a reputation of being of a higher accuracy; however, they may also be affected by slight systematic over- or underestimation of intensities.

For either X-ray or neutron diffraction experiments, small atomic displacement parameters may arise from effects that artificially increase *I*_obs_ > *I*_Bragg_ and thus may just be an artefact. Systematic errors that artificially reduce atomic displacement parameters may need to be excluded in order to ensure that small atomic displacement parameters are physically meaningful and not an artefact in X-ray and neutron diffraction experiments.

## Figures and Tables

**Table 1:** Distribution of selected characteristics of the data set, including minimum, maximum, mean and median values for the number of reflections used in the least-squares minimization (*N*_obs_), the weighting-scheme parameters *a* and *b*, the number of refined model parameters (*N*_param_), the fraction (*N*_obs_ − *N*_param_)/*N*_obs_, the maximum crystal size (in mm), the average *R* factor for equivalent reflections, the conventional *R* factor, and the weighted agreement factor Data sets processed using *SADABS*† (6781), Rigaku software‡ (1091), *SORTAV* (232), Stoe & Cie software (235) or unspecified software (85).

	Minimum	Maximum	Mean	Median
*N*_obs_	314	38082	3534.62	3007.00
*a*	0.0000	0.2400	0.0633	0.0591
*b*	0.0000	38.7799	0.4993	0.2171
*N*_param_	35	3345	243.58	214.00
(*N*_obs_ − *N*_param_)/*N*_obs_	0.5617	0.9916	0.9253	0.9290
_exptl_crystal_size_max	0.0300	10.280	0.3607	0.3300
_diffrn_reflns_av_R_equivalents	0.0000	0.3610	0.0426	0.0352
_refine_ls_R_factor_all	0.0182	0.3024	0.0759	0.0685
_refine_ls_wR_factor_ref	0.0483	0.3664	0.1324	0.1260

† All releases including *SADABS* 1996, with the largest fraction of 1919 data sets.

‡ Includes releases from Agilent and Oxford Diffraction: *CrysAlis PRO* Agilent 2010, *CrysAlis PRO* Agilent 2011, *CrysAlis PRO* Agilent 2012, *CrysAlis PRO* Agilent 2013, *CrysAlis PRO* Agilent 2014, *CrysAlis PRO* Oxford Diffraction 2009, *CrysAlis PRO* Oxford Diffraction 2010, *CrysAlis PRO* Rigaku OD 2015, *CrysAlis RED* Oxford Diffraction 2006, *CrysAlis RED* Oxford Diffraction 2007, *CrysAlis RED* Oxford Diffraction 2008, *CrysAlis RED* Oxford Diffraction 2009, *CrystalClear* Rigaku MSC 2005, *CrystalClear* Rigaku 2005.

**Table 2:** Summary of the simulated data sets, including the increment *n* for the applied offset leading to the total offset δ_SIM_; maximum, minimum and average increments relative to σ(*I*_obs_); the total change relative to 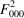; the resulting weighting-cheme parameters *a* and *b*; minimum and maximum simulated intensities; mean weighted residuals; and the significance of the mean weighted residuals The weighting-scheme parameters remain zero even for highly significant deviations, as the applied offset results in many small residuals rather than large residuals.

	*n*	δ_SIM_	[δ_SIM_/σ(*I*_obs_)]_max_	[δ_SIM_/σ(*I*_obs_)]_min_	[δ_SIM_/σ(*I*_obs_)]_av_	*N*_obs_δ_SIM_/*F*_000_^2^	*a*	*b*	(*I*_SIM_)_min_	(*I*_SIM_)_max_	〈ζ〉	〈ζ〉/σ(〈ζ〉)
SIM 14	−8	−0.0032*F*_000_	−0.0001	−1.8420	−0.1929	− 0.0163	0.00	0.00	−5.81	9899.20	−0.1117	−7.7025
SIM 18	−4	− 0.0016*F*_000_	−0.0001	−0.9210	−0.0964	− 0.0081	0.00	0.00	−5.69	9899.31	−0.0566	−3.9511
SIM 22	0	0.0000*F*_000_	0.0000	0.0000	0.0000	0.0000	0.00	0.00	−5.58	9899.42	−0.0019	−0.1321
SIM 26	4	0.0016*F*_000_	0.9210	0.0001	0.0964	0.0081	0.00	0.00	−5.47	9899.53	0.0527	3.6798
SIM 30	8	0.0032*F*_000_	1.8420	0.0001	0.1929	0.0163	0.00	0.00	−5.36	9899.64	0.1071	7.3924

## Footnotes

1 Within certain limits: The more uniform the distribution of δ_*j*_ values for a given set of redundantly measured intensities  is, the more exactly is the sum in equation (5) invariant. Equation (5) holds exactly in the case of a calibration error, where basically the origin has the same offset δ for all reflections. For larger differences between individual values of δ_*j*_, the described cancellation with the mean value does not work fully anymore, but the equation holds approximately when the set of δ_*j*_ is sufficiently uniform.

## Appendix A The simulations

The data set collected by Shraddha *et al.* (2020 ▸) was selected from the sample. It describes a structure crystallizing in the monoclinic space group *P*2_1_/*n* and contains a moiety belonging to the imidazoles (C_29_H_23_ClN_2_O). The measurement was conducted on a Bruker diffractometer with Mo *K*α radiation at 297 K.

For preparing the simulation, first the refinement was repeated with the command OMIT -100 but no other changes. This ensures that all reflections are taken into account, including large negative intensity observations. From the resulting reflection list file, the calculated intensities were extracted. They correspond to the true Bragg intensity without any noise. For the reference simulation SIM 22, noise was added to each reflection in proportion to the s.u.(*I*_obs_) from the experimental data. The model parameters resulting from this reference refinement are defined to be the true model parameters and correspond to a refinement against observed intensities unbiased with respect to the true intensities as described in equation (2).

For refinements including a systematic shift δ of the origin like for a calibration error, equation (3), *n* increments of δ_SIM_ = ±0.0004*F*_000_ were added to all *I*_true_ and exactly the same noise was added. For example, when the noise was +1.234 for reflection 234 in SIM 22, it was also +1.234 in all other simulations (SIM 14, SIM 18, SIM 26, SIM 30). This is to make sure that the results do not depend on the set of random numbers. In order to monitor the effect on the individual reflection, the maximum, minimum and mean value for δ_SIM_/σ(*I*_obs_) was calculated for each individual reflection in each simulated data set. The aim was to make the added systematic error insignificant with respect to σ(*I*_obs_) such that it would not give rise to large residuals. SIM 22 is the reference simulation with no systematic error, δ_SIM 22_ = 0. Simulations with numbers >22 have the increment added, while the others have it subtracted. For example, in SIM 26, δ_SIM 26_ = +0.0016*F*_000_ for each reflection (*n* = 4 as 26 − 22 = 4), and in SIM 30 δ_SIM 30_ = +0.0032*F*_000_. The largest change for an individual reflection in SIM 30 due to the systematic error was (δ_SIM 30_)_max_ = 1.8420σ(*I*_obs_), well below three standard deviations of the observed intensity. Similarly, for SIM 14, the largest change was negative, δ_SIM 14_ = −1.8420σ(*I*_obs_).

The average change due to systematic errors was a reduction of the individual reflections by 0.1929σ(*I*_obs_) for SIM 14 and correspondingly an increase by 0.1929σ(*I*_obs_) for SIM 30. This corresponds for SIM 30 to adding to all reflections in a uniform way the total amount of additional scattering mass of 1.63% of , which leaves the relative maximum simulated intensities virtually unaffected when compared with the true value from SIM 22. This leads to maximum changes in the weakest reflections by approximately only 4% when compared with the true value of SIM 22. Overall it can be said that the changes induced by the simulation are all small and most likely much smaller than other errors, for example due to a fluctuating beam intensity. The weighting scheme parameters *a* and *b* remain consequently zero after invoking the weighting scheme. Despite each individual distortion from the true intensity being small, the overall effect of these many small but one-sided distortions adds up to a measurable effect (Table 2 ▸).

A shift of the weighted residuals by 0.0527 like in SIM 26 may appear small; however, it is already significant at 3.6798σ. The significance is given by dividing the mean value by the standard deviation of the mean value, , where  is the unbiased sample variance. The larger *N*_obs_, the smaller the standard deviation of the mean value. The simulations SIM 14–SIM 30 all have the same *N*_obs_ = 4807. 〈*N*_obs_〉 = 3534.62 for the 8424 data sets from the sample described in Section 2.
